# Long‐distance dispersal by a male sub‐adult tiger in a human‐dominated landscape

**DOI:** 10.1002/ece3.9307

**Published:** 2022-09-27

**Authors:** Zehidul Hussain, Pallavi Ghaskadbi, Pramod Panchbhai, Ravikiran Govekar, Parag Nigam, Bilal Habib

**Affiliations:** ^1^ Wildlife Institute of India Chandrabani, Dehradun India; ^2^ Maharashtra Forest Department Maharashtra India

**Keywords:** carnivore, displacement, habitat fragmentation, natal area, telemetry

## Abstract

Conservation of wide‐ranging species and their movement is a major challenge in an increasingly fragmented world. Long‐distance movement, such as dispersal, is a key factor for the persistence of population, enabling the movement of animals within and between populations. Here, we describe one of the longest dispersal journeys by a sub‐adult male tiger (*Panthera tigris*) through GPS telemetry in Central India. We analyzed movement metrics, directionality, and space use during three behavioral stages of dispersal. We also used the clustering method to identify resting and kill sites (*n* = 89). T1‐C1 dispersed a straight‐line distance of 315 km over 225 days, moving an average of 8.38 km/day and covering a cumulative displacement of ~3000 km. Movement rate during post‐dispersal was faster (mean = 0.47 km/h) than during dispersal (mean = 0.38 km/h) and pre‐dispersal (mean = 0.13 km/h), respectively. The overall movement rate during the night (0.44 km/h) was significantly faster than during the day (0.21 km/h). Likewise, during dispersal, the movement was faster (mean = 0.52 km/h) at night than day (0.24 km/h). The average size of clusters, signifying resting and kill sites, was 1.68 ha and primarily away from human habitation (mean = 1.89 km). The individual crossed roads faster (mean = 2.00 km/h) than it traveled during other times. During the post‐dispersal phase, T1‐C1 had a space use of 319.48 km^2^ (95% dBBMM) in the Dnyanganga Wildlife Sanctuary. The dispersal event highlights the long‐distance and multiscale movement behavior in a heterogeneous landscape. Moreover, small forest patches play a key role in maintaining large carnivore connectivity while dispersing through a human‐dominated landscape. Our study underlines how documenting the long‐distance movement and integrating it with modern technology can improve conservation management decisions.

## Graphical abstract

The article is about the longest known dispersal by a sub‐adult tiger in a human‐dominated landscape.

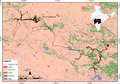

## 1 INTRODUCTION

Habitat loss and increased human land use are primary threats to many wildlife species, altering their movement and behavior (Tucker et al., 2018). Animal movement is further reduced by habitat fragmentation, degrading habitat quality and linear features such as roads (Andersen et al., 2017; Crooks et al., 2017; Fahrig, 2003; Jønsson et al., 2016; Shepard et al., 2008). Mammalian carnivores tend to have large home ranges and long dispersal distances, making them vulnerable to landscapes that have been fragmented by anthropogenic changes (Crooks et al., 2011; Dobson et al., 2006; Ripple et al., 2014). Long‐distance dispersal is central to several ecological processes (Levin et al., 2003; Nathan, 2003), including gene flow (Trakhtenbrot et al., 2005), colonization of new areas, range shift, and functional connectivity between populations (Clobert et al., 2012; Noss et al., 1996; Weaver et al., 1996). The long‐term population persistence in fragmented landscapes may depend on individuals traversing through a human‐dominated landscape to reach suitable habitats (Watts et al., 2015).

In India, protected areas (PA) designated as tiger reserves cover 2.21% of the geographical region, and 35% of the total tiger population resides outside these PAs (Habib, Ghaskadbi, et al., 2021). The tiger populations are mostly confined to small protected areas because the habitat outside those areas is highly fragmented, which affects their movement within and outside protected areas (Habib, Nigam, et al., 2021; Thatte et al., 2018). Consequently, it is challenging for tigers to move through a fragmented and human‐dominated landscape. Moreover, the long‐term survival of the tiger population in a fragmented landscape depends on successful dispersal from one area to another, thus maintaining the connectivity between subpopulations and isolated populations.

Dispersal involves three successive behavioral stages: departure, transience, and settlement (Clobert et al., 2009). Accordingly, the dispersal behavior of sub‐adult tigers can be classified into three distinct behavioral phases, that is, pre‐dispersal, dispersal, and post‐dispersal. The pre‐dispersal phase is identified by the movement of individuals within their natal area. The dispersal phase involves movement from its area of birth to another habitat, where it reproduces and establishes a new territory (Howard, 2015; Waser & Jones, 1983). Post‐dispersal phase is identified by movement in an area having a stable and defined home range over time. The movement behavior during dispersal in a highly interspersed mosaic of forested areas, agriculture fields, and human settlements is rarely documented. Studies on tiger dispersal are limited, and few have described long‐distance dispersal primarily based on camera trap data and VHF/GPS telemetry (Sarkar et al., 2021; Singh et al., 2021; Smith, 1993; Wang et al., 2015). Moreover, genetic studies have shown gene flow over long distances (Anuradha Reddy et al., 2016; Gour et al., 2013; Joshi et al., 2013).

The advancement of GPS technology made it possible to gain new insights into tiger dispersal and how animals perceive and navigate through a landscape. Additionally, long‐distance dispersal routes might help identify land use and landscape features that provide connectivity between non‐contiguous populations (Graves et al., 2007). This study reports the longest dispersal distance recorded by a sub‐adult male tiger from the Vidarbha region of Maharashtra, India. We documented movement and space use from the pre‐dispersal to the post‐dispersal phase and described the chronological event during dispersal. We analyzed the movement data from the GPS collar with environmental covariates (land use and vegetation cover) depicting the landscape through which the individual moved. Our study also quantified the characteristics of clusters (resting/kill) and the effect of linear features, that is, roads, on movement. We conclude by discussing the long‐distance dispersal in a human‐dominated landscape and its implications for conservation and management.

## 2 MATERIAL AND METHODS

### 2.1 Study area

The study was carried out within and outside of protected areas of Eastern Vidarbha Landscape (EVL), Maharashtra, India. This region is a part of the Central Indian Tiger Landscape, dominated by teak (*Tectona grandis*) and bamboo (*Dendrocalamus strictus*). The region encompasses an area of approximately 97,320 km^2^, and forest cover accounts for 27.5% of the total area (Habib, Nigam, et al., 2021). The landscape is a mosaic of agricultural lands, human settlements and wildlife areas (Habib et al., 2017). Large carnivores in the region include tiger (*Panthera tigris*), which co‐occurs with other species like leopard (*Panthera pardus*), sloth bear (*Melursus ursinus*), gaur (*Bos gaurus*), and several other ungulate species.

### 2.2 Field methods

We captured and collared sub‐adult male tiger T1‐C1 on February 25, 2019. The individual was immobilized using a combination of Medetomidine hydrochloride, Ketamine hydrochloride, and Xylazine. The dosages were based on visual observation of body weight (150 kg) and age. The drug was remotely injected using an air‐pressurized Dan‐Inject projector (Model IM). The radio‐collaring was part of a more extensive study in which sub‐adult tigers were collared to understand the dispersal patterns, space use, and movement in a human‐dominated landscape. Tigers were classified as sub‐adult following (Sadhu et al., 2017). Animals were fitted with Iridium GPS radio‐collars (Vectronics, Germany) and had a high spatial accuracy of location (±5 m). The collar was programmed to record locations every 1–3 h depending on the dispersal behavior of the individual. During the pre‐dispersal phase, when the tiger was within its natal area, we received location every 3‐h interval. During dispersal, when it moved out of its natal area, we programmed to receive intensive location every 1‐h interval. During the post‐dispersal phase, locations were received every 3‐h time interval.

### 2.3 Analysis

We calculated movement metrics (movement speed and turning angle) within each of three phases: pre‐dispersal, dispersal, and post‐dispersal. We identified these three phases by calculating the net squared displacement (NSD) in ArcGIS 10.6.1 with ArcMET tool (Wall, 2014). NSD is movement metrics that helps to understand movement behaviors over time (Bunnefeld et al., 2011). Inflection points and increase in the NSD over time help identify movement modes such as migration, dispersal, nomadism, and range residency (Börger & Fryxell, 2012). We identified dispersal movement and age from the peak in the NSD graph, which indicates the commencement of dispersal from its natal area.

We calculated daily distance traveled (sum of displacement in 1 day) and daily displacement (linear distance between the start and end locations for each 24‐h period) during all three phases. We considered a 24‐h period from the first location of dawn and the first location of the following dawn. We also estimated the mean movement speed (km/h) across the three dispersal phases. To calculate the movement speed, we scaled the step length (Euclidean distance between successive locations) divided by the time the individual took to complete the distance due to the varying interfix intervals (Leblond et al., 2016). The movement parameters were calculated using adehabitatLT (Calenge, 2015) and the animal movement tool (Signer et al., 2019) in the R programming software (R Core Team, 2020). We used the Kruskal–Wallis test to compare the mean movement speed during the pre‐dispersal, dispersal, and post‐dispersal phases as data were not normally distributed. Next, we used the Mann–Whitney *U* test to compare day and night movement speeds. To estimate the space use in each of the three phases, we applied the dynamic Brownian Bridge Movement Model (dBBMM) to estimate the utilization distribution in the package move (Kranstauber et al., 2012). The dBBMM requires a time‐stamped series of animal locations and the estimated telemetry error associated with each location. The dBBMM allows the variance of the Brownian motion (*σ2m*) to vary along the movement path for user‐defined subsets of *n* locations. We then included these values in a Brownian bridge movement model to estimate 95% utilization distributions (UDs). We considered the 95% utilization distribution as space use in three dispersal phases.

To understand the directionality or orientation of the animal, we calculated the turning angles across the three phases. We estimated the circular mean, from the von Mises distribution of tuning angles (Mardia & Jupp, 1999). The mean turning angle is between −π and π and defines the degree of linearity in an animal movement. A mean turning angle of zero implies a strong persistence in direction, whereas a mean turning angle of −π or π suggests that movement is undirected. We used the package circular (Lund et al., 2017) to calculate the circular mean and test for directionality. All calculations and statistical analysis were carried out in program R 4.0.4 (R Core Team, 2020).

We identified resting and kill clusters formed during dispersal using the GPSeqClus package (Clapp et al., 2021). The algorithm uses time‐series location data to sequentially aggregate locations to build clusters based on three user‐defined criteria: search radius, temporal window, and minimum number of locations. We used the average step length per hour of T1‐C1 as the search diameter for cluster identification. The maximum temporal window for cluster identification was 3 days, and we considered eight locations as minimum cluster locations. Resting and kill sites were validated from field observation. We calculated average cluster size, time spent, number of visits, and distance to the nearest forest edge and human settlement. To estimate habitat composition, we created a buffer of mean cluster radius around every cluster's median location and extracted the percentage of land cover in ArcGIS (10.6.1). The land use data of 60 m resolution were acquired from Bhuvan's open‐source website (NRSA, 2016; http://bhuvan.nrsc.gov.in/). We also used normalized difference vegetation index (NDVI) to indicate vegetation cover of the resting clusters and kill sites. NDVI datasets from Landsat 8 were obtained from Google Earth Engine™ (GEE) between June 2019 and January 2020 at a spatial and temporal resolution of 30 m and 32 days, respectively (Gorelick et al., 2017). From the start date of each cluster, the nearest available mean NDVI was generated. NDVI value of one indicates a high density with green leaves, and a negative value indicates no vegetation.

## 3 RESULTS

**FIGURE 1:**
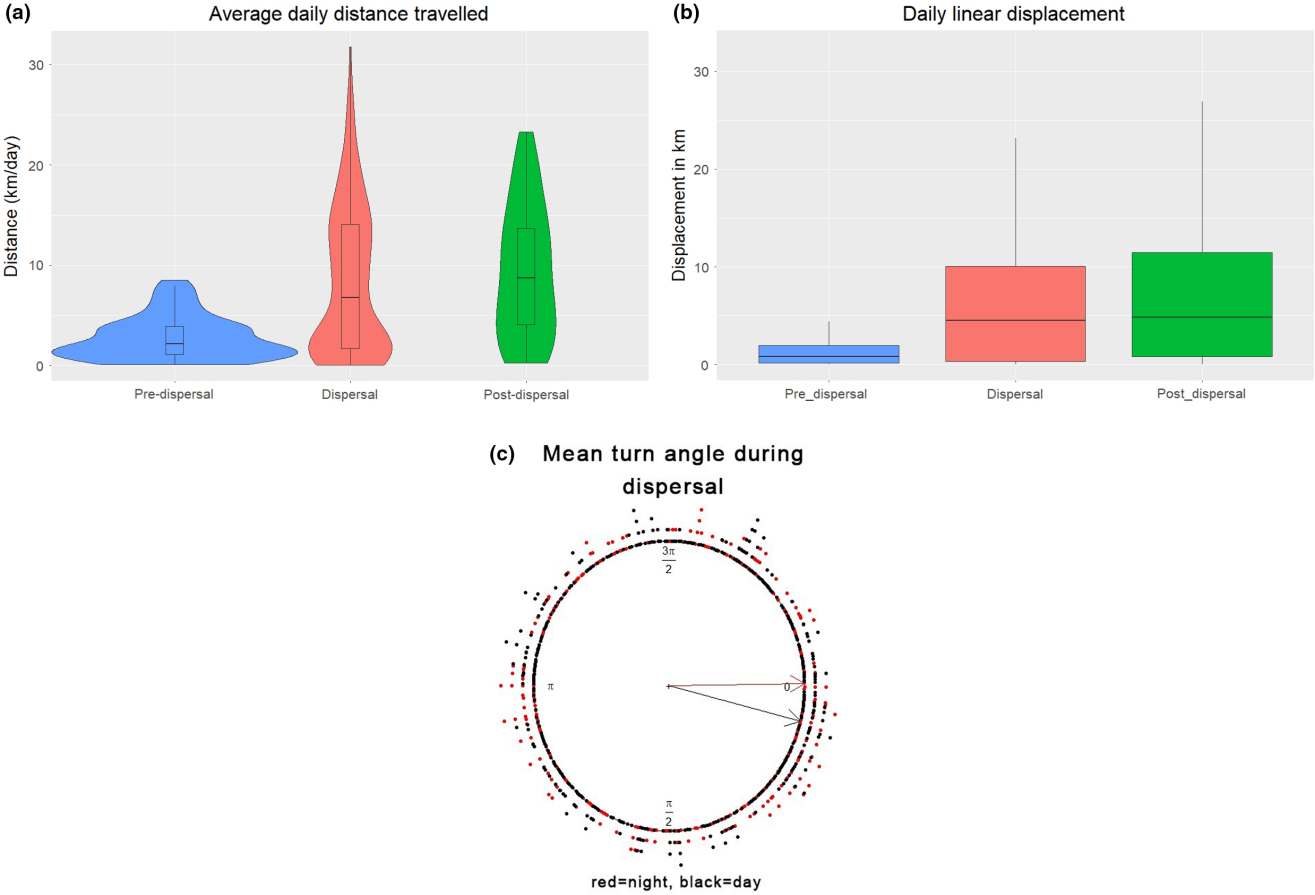
Movement of a sub‐adult tiger (*Panthera tigris*) T1‐C1 during pre‐dispersal, dispersal, and post‐dispersal phase in the Vidarbha Landscape of Maharashtra, India (a) average daily distance traveled, (b) daily linear displacement, and (c) mean turn angle during dispersal (red = night, black = day).

T1‐C1 was tracked for 396 days and recorded 6240 GPS locations between February 2019 and March 2020. We describe the movement, exploration, and space use during the pre‐dispersal, dispersal, and post‐dispersal period. We report the chronological events that occurred during the dispersal. The space use during the pre‐dispersal phase in the Tipeshwar Wildlife Sanctuary covered an area of 18.72 km^2^ (95% dBBMM). The average daily distance covered was 2.76 km (range: 0.10–8.52 km), linear displacement of 1.33 km/day, and traversed maximum displacement of 5.46 km in a day (Figure 1a,b). The dispersal event spanned 225 days from June 21, 2019, to January 31, 2020, within a human‐dominated landscape. T1‐C1 traversed 2000 km by moving an average daily distance of 8.38 km (range: 0.05–31.75 km) and linear displacement of 6.19 km/day and covering a maximum daily displacement of 32.53 km. During dispersal, the utilization area was 1077.98 km^2^ (95% dBBMM). The post‐dispersal phase was identified by stable and territorial movement in the Dnyanganga Wildlife Sanctuary and covered an area of 319.48 km^2^ (95% dBBMM). During this phase, it traveled an average daily distance of 9.29 km (range: 0.25–23.31 km) and linear displacement of 6.91 km/day (range: 0.003–26.31 km). The straight‐line displacement between the natal area and the furthest location was approximately 315 km.

### 3.1 Movement speed

The mean movement speed exhibited by T1‐C1 was highest during post‐dispersal (0.47 km/h; range: 0–3.26 km/h). During the dispersal and pre‐dispersal period, the average movement speed was 0.38 km/h (range: 0–3.50 km/h) and 0.13 km/h (range: 0–2.5 km/h), respectively (Table 1). During the pre‐dispersal phase, there was no significant difference in the mean movement speed during the day (0.11 km/h; range: 0–2.56 km/h) and at night (0.15 km/h; range: 0–2.29 km/h). While dispersing, the average movement rate varied significantly between day (0.24 km/h, range: 0–2.36 km/h; *p* < .001) and night (0.52 km/h, range: 0–3.50 km/h). Similarly, mean hourly displacement at night (0.65 km/h; range: 0–3.26 km/h) was also more than day (0.27 km/h; range; 0–2.15 km/h) during the post‐dispersal period (*p* < .001).

**TABLE 1:** Mean movement speed of a sub‐adult tiger (*Panthera tigris*) T1‐C1 during pre‐dispersal, dispersal, and post‐dispersal phase from the Vidarbha Landscape of Maharashtra, India.

Phase	Movement (km/h)		
	Overall (min–max)	Day (min–max)	Night (min–max)
Pre‐dispersal	0.13 (0–2.56)	0.11 (0–2.56)	0.15 (0–2.29)
Dispersal	0.38 (0–3.50)	0.24 (0–2.36)	0.52 (0–3.50)
Post‐dispersal	0.47 (0–3.26)	0.27 (0–2.15)	0.65 (0–3.26)

### 3.2 Directionality

During the pre‐dispersal phase, the movement of T1‐C1 was undirected (mean turning angle = −2.88) suggesting uniform distribution of turning angle. During the dispersal, the mean turning angle was 0.11, indicating strong persistence in direction and linear movement. Moreover, the directional persistence or tendency to move in a straight line was more at night (mean turning angle = −0.01) than during the day (mean turning angle = 0.24; Figure 1c). During post‐dispersal, the wide range in the mean turning angle (mean_day_ = −0.60; mean_night_ = 0.24) indicates a mix of tortuous and forward movement in the landscape.

### 3.3 Chronological dispersal event

The dispersal event of T1‐C1 occurred between two protected areas, Tipeshwar and Dnyanganga Wildlife Sanctuary, separated by a linear distance of 240 km. The movement during dispersal occurred in a landscape with a mosaic of tropical dry deciduous forest, open scrub, agricultural fields, and human habitation. T1‐C1 began dispersing from the natal site, that is, Tipeshwar Wildlife Sanctuary, on June 21, 2019, at the age of 30 months. The density of tigers at the Tipeshwar WLS was reported 6.18 individuals/100 km^2^ (Habib et al., 2020). T1‐C1 initial movement was confined to a small plantation in a nursery adjacent to the park. During this period, there were two resting clusters, one within the plantation and the other 800 m away. After 12 days, the tiger started moving in the southern direction crossing the Painganga river into the state of Telangana. The forest patches that are located southward act as a wildlife corridor and connect to nearby protected places like the Painganga Wildlife Sanctuary and, further south, Kawal Tiger Reserve.

**FIGURE 2:**
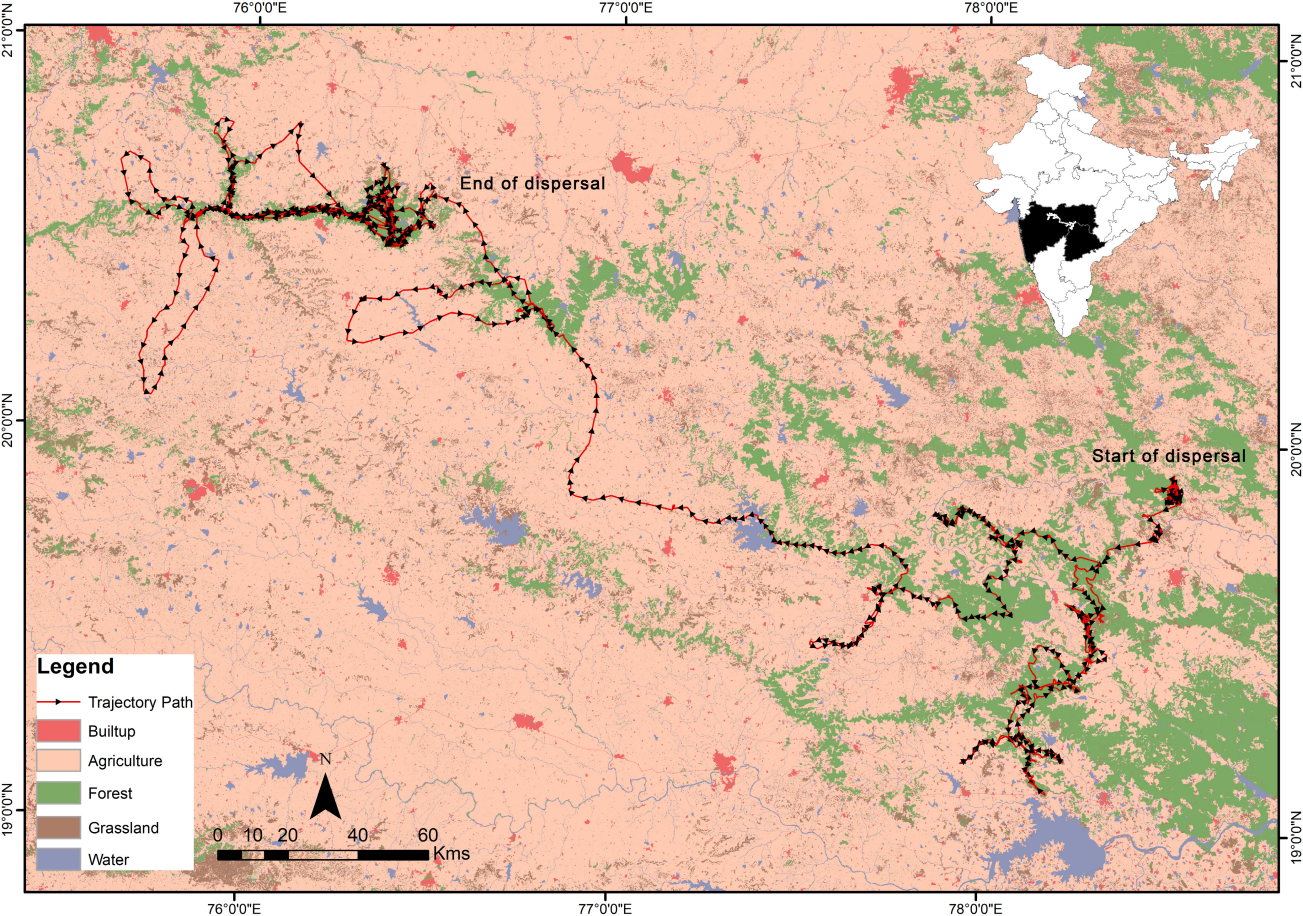
Dispersal movement of a sub‐adult tiger (*Panthera tigris*) T1‐C1 from Tipeshwar to Dnyanganga Wildlife Sanctuary traveling through a human‐dominated landscape.

T1‐C1 continued southward movement through the corridor, crossing a primary road (*n* = 1) and minor roads (*n* = 20). The southward trajectory was maintained until 12 July, covering a linear distance of 200 km from its natal area. However, T1‐C1, on encountering a small village and an adjacent 4‐lane national highway, turned and continued moving north along the same corridor path (Figure 2). This northward movement continued until 22 September and moved west along which the corridor extends. This westward trajectory movement continued for another 100 km, and then the tiger turned around and retraced his steps in the east direction. However, after traveling for a straight‐line distance of 50 km, the tiger changed direction and moved toward the south during October 10–12, 2019.

The trajectory of T1‐C1 after 12 October was west and northwestern direction moving through a mosaic of fragmented forest patches and primarily dominated by agricultural fields until it reached another forested area. It took him 25 days to get to this area after moving through a landscape with high human population, roads and railway lines. Until 27 November, the tiger explored the forested landscape with wild prey and domestic cattle kills and finally moved north to enter the Dnyanganga Wildlife Sanctuary. T1‐C1 entered the sanctuary on 29 November, where he later established his area. However, before establishing a stable area in the sanctuary, he moved further 90 km west and southwest of the sanctuary, including the forested landscape of the Ajanta caves. After more than a month of exploring the landscape, T1‐C1 movement was confined to the Dnyanganga Sanctuary and adjoining fragments of forest patches outside the PA and eventually had a defined core area within the sanctuary. We monitored the individual through camera trapping in the sanctuary for 10 months after the collar was removed. There was no report of the presence of tigers other than T1‐C1 when it entered the sanctuary. However, co‐predator like leopards was present in the area and adjoining forest patches.

**FIGURE 3:**
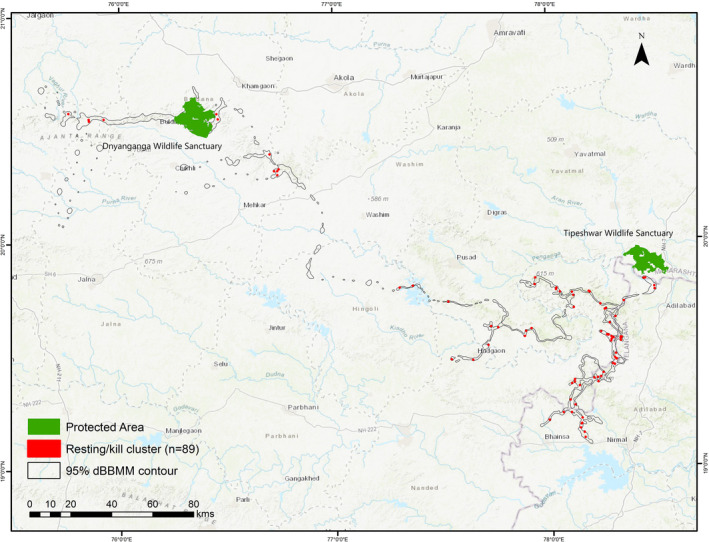
Resting clusters and kill sites of a sub‐adult tiger (*Panthera tigris*) T1‐C1 during dispersal in a human‐dominated landscape. The dispersal direction is from Tipeshwar to Dnyanganga Wildlife Sanctuary.

Overall, the cumulative distance traveled by T1‐C1 was 3000 km. During the dispersal phase, it traveled 2000 km and covered eight districts of Maharashtra and two states of India between June 2019 and January 2020. While traversing through a mosaic of forested and agricultural landscapes, we identified 89 clusters (forest = 73; outside forest = 16), of which 12 were known kill sites (Figure 3). The average size of the cluster was 1.68 ha (range: 0.001–10.74 ha; *n* = 89) in a human‐altered landscape. The mean cluster size inside forest (1.91 ha; range: 0.01–10.74 ha) was significantly larger than in non‐forested areas (0.62 ha; range: 0.001–3.30 ha; *p* < .05). The average cluster duration was less in the forest (38.41 h; range: 7–122 h) than in areas outside (46.31 h; range: 7–176 h). Moreover, T1‐C1 spent more time in clusters with larger sizes (forest: 6–8 ha and outside forest: 2–4 ha); in both forest and non‐forest areas (Table 2). The average cluster distance inside the forest was 2 km (range: 0.3–5.0 km) from human settlements, while in non‐forested areas, it was 1.4 km (range: 0.52–2.5 km). The average NDVI value of clusters was 0.23 and ranged from 0.12 to 0.38. Overall, clusters had the highest proportion of forest (83.4%), followed by agriculture (11.4%), grassland (4.6%) and water (0.5%), respectively.

**TABLE 2:** Cluster characteristics (cluster size, number of clusters, time duration, and distance to human settlements) of a sub‐adult tiger (*Panthera tigris*) T1‐C1 while dispersing through a human‐dominated landscape in the Vidarbha Landscape of Maharashtra, India

System	Cluster size (ha)	Number of clusters	Mean time spent (h) ± SD	Number of visits	Mean NDVI ± SD	Distance to forest edge (m) ± SD	Distance to human settlement (m) ± SD
Forest	0–2	43	32.98 ± 25.66	2	0.23 ± 0.05	433.07 ± 357.19	1898.78 ± 1159.15
	2–4	22	47.36 ± 28.18	2	0.24 ± 0.05	438.46 ± 311.19	2207.30 ± 778.83
	4–6	4	20.75 ± 12.61	2	0.21 ± 0.07	606.77 ± 444.59	1302.89 ± 687.84
	6–8	3	68.00 ± 52.12	4	0.20 ± 0.04	223.30 ± 257.54	2150.23 ± 394.60
	>8	1	57.00	3	0.26	1279.93	3337.51
Non‐forest	0–2	14	42.43 ± 57.35	2	0.20 ± 0.05	1245.77 ± 1607.74	1345.46 ± 737.41
	2–4	2	73.50 ± 94.05	3	0.17 ± 0.02	1238.92 ± 4.74	1441.57 ± 600.36

While dispersing through a human‐dominated landscape, T1‐C1 crossed linear features such as railway lines (*n* = 7), interstate highways (*n* = 34), and primary roads (*n* = 67) before reaching the Dnyanganga Sanctuary. The individual crossed primary roads more at night (*n* = 51) than day (*n* = 16). Similarly, it crossed highways at night (*n* = 29) more often than day (*n* = 5) while dispersing in a human‐dominated landscape. The movement rate significantly increased when crossing roads (mean = 2.00 km/h) than traveling in non‐road areas (mean = 0.30 km/h; *p* < .05). Subsequently, the trajectory path of T1‐C1 showed territorial movement behavior confined to the sanctuary and adjoining forested landscape. Therefore, after tracking for more than a year, the GPS radio‐collar was removed using a drop‐off mechanism.

## 4 DISCUSSION

We recorded one of the longest dispersals of a male tiger, traveling a maximum linear distance of 315 km in a human‐dominated landscape. The dispersal journey was remarkable for its length, duration, and movement through a highly interspersed mosaic of forested areas and agricultural fields. Evidence of such long‐distance dispersal in tigers has been recorded previously in a few studies through radiotelemetry (VHF and GPS), intensively monitored populations (camera traps) and genetic studies across various landscapes in the Indian subcontinent (Gour et al., 2013; Reddy et al., 2012; Sarkar et al., 2016, 2021; Singh et al., 2021; Smith, 1993). Such movement occurs in both sexes, with males moving longer distances from few kilometers to hundreds of kilometers (Table 3) and can be influenced by various factors. In general, male dispersal is related to intrasexual competition for mates, inbreeding avoidance, and resource competition (Dobson, 1982; Greenwood, 1980; Perrin & Vladimir, 1999; Pusey & Wolf, 1996). Information from long‐distance dispersal thus provides knowledge and helps identify functional corridors, which are crucial to designing conservation policies for large‐ranging species like tigers.

**TABLE 3:** Details of dispersal studies on tigers in the Indian subcontinent and distance traveled by males from their natal area during dispersal

Author	Place	Method	Euclidean dispersal distance (km)	Cumulative distance covered (km)
Smith (1993)	Royal Chitwan National Park, Nepal	VHF collar	71	–
Sarkar et al. (2016)	Panna Tiger Reserve	VHF/GPS/Satellite collar	250	440
Murthy (2017)	Bandhavgarh Tiger Reserve	Camera trapping	280	–
Sadhu et al. (2017)	Ranthambhore Tiger Reserve	Camera trapping	220	–
Singh et al. (2021)	Ranthambhore Tiger Reserve	Camera trapping	148.4	–
Current study (T1‐C1)	Vidarbha Landscape	GPS collar	315	~3000

The dispersal journey of T1‐C1 involved three distinct movement phases and traveled a cumulative distance of ~3000 km from its natal area to the site where it localized after dispersal. The dispersal route of a large‐ranging carnivore like tigers highlights the multiscale nature of the individual movement and the ability to navigate a heterogeneous landscape. The dispersal event started during the monsoon and lasted until winter. During the period of monsoon, vegetation cover, availability of water, and productivity in dry deciduous habitat increases, making dispersal more permeable through a human‐dominated landscape. Moreover, the agricultural fields with growing crops act as a cover for dispersal. Additionally, the availability of livestock increases as they are left to graze in the forested areas and form easy prey for dispersing tigers.

During post‐dispersal, we found that the daily distance traveled and displacement was higher than in the dispersal and pre‐dispersal phases. We also found a wide range in turning angles, suggesting the movement to be more tortuous and forward movement. This is probably because of the environmental features and mosaic of habitats within the landscape that influences movement. T1‐C1 space use included multiple core areas (17.29 km^2^) within the Dnyanganga Sanctuary and encompassed a larger fragmented area, including forest and agricultural fields outside the PA. Thus, it adapted a high territorial movement to cover a more extensive territory while crisscrossing agriculture fields and forested areas in a human‐dominated landscape. Similarly, studies on cougars and lions exhibited higher speeds while traversing through fragmented human‐dominated areas to reduce time spent in multiple‐use areas (Kertson et al., 2011; Valeix et al., 2012). Moreover, temporal changes in movement speed allow the individual to cover longer distances and more fragmented forest patches occurring both inside and outside the sanctuary. Thus, it minimizes the anthropogenic disturbances by moving more at night (0.65 km/h) than during the day in a human‐dominated landscape. Similar movement behavior was reported in tigers that exhibited higher speeds outside PA and at night (Habib, Ghaskadbi, et al., 2021).

The movement was more linear with strong directional persistence during dispersal with a maximum mean movement speed of 3.50 km/h during the night. Altered movement patterns due to habitat fragmentation and human pressure have been observed in many carnivore species worldwide (Poessel et al., 2014; Tigas et al., 2002; Tucker et al., 2018). His movement was more confined and restricted within small forest patches and sheltered in these patches during the day (mean turn angle = 0.24). While at night, it traveled extensively from one forest patch to another interspersed in the landscape with high directional persistence (mean turn angle = −0.01). This variability in movement behavior can be attributed to behavioral plasticity while moving through a human‐dominated landscape, thus minimizing human encounters and reducing anthropogenic pressure. Moreover, change in land use and increase fragmentation limits the movement of dispersing tigers. To negotiate movement in such a human‐dominated area, animals move faster at night with more directionality (Habib, Ghaskadbi, et al., 2021; Ordiz et al., 2017).

The clusters formed along the dispersal path highlighted the characteristics of resting places or short‐term refugia and also acted as kill sites, where it persisted mainly on domestic cattle. Our result shows that T1‐C1 spent less time per event in larger clusters inside the forest and more time in smaller clusters outside forested areas. The differential cluster size and time spent in non‐forest areas with respect to forested areas can be attributed to the mosaic habitat and anthropogenic disturbances. The habitat patches in a human‐dominated landscape are at varying distances from each other and interspersed with agricultural fields. Due to the fragmented habitat outside forested areas, small clusters were observed between forest fragments as probable resting sites. Such clusters were away from human settlements, primarily in moderate‐to‐high vegetation cover. However, the time spent in these small clusters was higher as the animal would be compelled to rest in these refuge patches during the daytime when human activity is maximum. As human activity reduces at night, the animal can move between forest fragments situated far away from each other. Whereas, in forested areas, the habitat is contiguous, making movement more conducive during day and night. Thus, the animal spent less time resting in larger clusters, perceiving a low risk attributed to human activity and anthropogenic disturbances.

In addition to the landscape heterogeneity, linear features such as roads affect the rate of movement while crossing roads compared to when traveling in non‐road areas. Moreover, T1‐C1 avoided high‐traffic roads by crossing roads at night as animal perceives roads and human activity as risk (Frid & Dill, 2002; Northrup et al., 2012; Thurfjell et al., 2015). Thus, linear features such as roads can act as barriers to animal movement either through mortality, displacement, or behavioral avoidance, depending on the landscape (Anderson, 2002; Forman et al., 2003; Scrafford et al., 2018; Shepard et al., 2008). Consequently, habitat mosaic and landscape structure greatly influence dispersal patterns by facilitating or restricting movement (Holderegger & Wagner, 2008).

### 4.1 Conservation implications

The study highlights the importance of documenting long‐distance dispersal by GPS telemetry, otherwise difficult to record at such a fine temporal scale. The dispersal event of T1‐C1 has interesting conservation implications as it moved through a human‐dominated landscape. While dispersing, the animal crossed numerous barriers such as rivers, national highways, major roads, and railway lines. The journey through a mosaic of land use revealed the significance of fragmented areas or refuge islands and dispersal corridors. The dispersal route provided the possible functional connectivity between the primary tiger habitat in Maharashtra and Telangana. However, the growing human population and further expansion of urban areas could compromise connectivity and influence movement. Because the tiger population relies upon dispersal between patches of primary habitat and adjoining landscape, land management and government agencies must consider enhancing connectivity and restoration of fragmented patches for the population's long‐term survival. The study also highlights the importance of small forest fragments in a landscape, which are essential in maintaining connectivity. Thus, successful animal dispersal indicates functional connectivity among habitats and can be used as an indicator for wildlife recovery.

## AUTHOR CONTRIBUTIONS

BH and PN conceived ideas and designed methodology; ZH carried out fieldwork, analyzed the data, and led the writing of the manuscript. PG helped in field data collection. RG and PP helped in capture and local field support. All the authors contributed critically to the draft and gave final approval for publication.

## FUNDING INFORMATION

Funding was provided by Maharashtra Forest Department, Government of Maharashtra.

## CONFLICT OF INTEREST

The authors declare that they have no competing interests.

## Data Availability

The dispersal data of the endangered species contain location data outside protected areas, which are prone to poaching and human prosecution. Moreover, the movement corridor and forested areas are used by other tigers and will make other dispersing individuals prone to many risks including poaching. Because of conservation reason, our request may be considered not to share the location data.
